# Promoter analysis of the rabbit *POU5F1 *gene and its expression in preimplantation stage embryos

**DOI:** 10.1186/1471-2199-10-88

**Published:** 2009-09-04

**Authors:** Julianna Kobolak, Katalin Kiss, Zsuzsanna Polgar, Solomon Mamo, Claire Rogel-Gaillard, Zsuzsanna Tancos, Istvan Bock, Arpad G Baji, Krisztina Tar, Melinda K Pirity, Andras Dinnyes

**Affiliations:** 1 Micromanipulation and Genetic Reprogramming Group, Agricultural Biotechnology Center, Szent-Györgyi A. u. 4. H-2100 Gödöllő, Hungary; 2 National Medical Center Cell Biology Department; Daróci u. 24. H-1113 Budapest, Hungary; 3 Faculty of Natural Sciences, Constantine the Philosopher University, Slovakia; 4 University College Dublin, Lyons Research Farm, Newcastle Co. Dublin, Ireland; 5 INRA, UMR1313, Laboratoire de Génétique Animale et Biologie Intégrative , 78350 Jouy en Josas , France; 6 Molecular Animal Biotechnology Laboratory, Szent Istvan University, Pater K. u. 1. H-2103 Gödöllő, Hungary; 7 BioTalentum Ltd., Aulich Lajos u. 26. H-2100 Gödöllő, Hungary

## Abstract

**Background:**

The *POU5F1 *gene encodes the octamer-binding transcription factor-4 (Oct4). It is crucial in the regulation of pluripotency during embryonic development and widely used as molecular marker of embryonic stem cells (ESCs). The objective of this study was to identify and to analyse the promoter region of rabbit *POU5F1 *gene; furthermore to examine its expression pattern in preimplantation stage rabbit embryos.

**Results:**

The upstream region of rabbit *POU5F1 *was subcloned sequenced and four highly conserved promoter regions (CR1-4) were identified. The highest degree of similarity on sequence level was found among the conserved domains between rabbit and human. Among the enhancers the proximal enhancer region (PE-1A) exhibited the highest degree of homology (96.4%). Furthermore, the CR4 regulator domain containing the distal enhancer (DE-2A) was responsible for stem cell-specific expression. Also, BAC library screen revealed the existence of a processed pseudogene of rabbit *POU5F1*. The results of quantitative real-time PCR experiments showed that *POU5F1 *mRNA was abundantly present in oocytes and zygotes, but it was gradually reduced until the activation of the embryonic genome, thereafter a continuous increase in *POU5F1 *mRNA level was observed until blastocyst stage. By using the XYClone laser system the inner cell mass (ICM) and trophoblast portions of embryos were microdissected and examined separately and *POU5F1 *mRNA was detected in both cell types.

**Conclusion:**

In this study we provide a comparative sequence analysis of the regulatory region of rabbit *POU5F1 *gene. Our data suggest that the *POU5F1 *gene is strictly regulated during early mammalian development. We proposed that the well conserved CR4 region containing the DE-2A enhancer is responsible for the highly conserved ESC specific gene expression. Notably, we are the first to report that the rabbit *POU5F1 *is not restricted to ICM cells only, but it is expressed in trophoblast cells as well. This information may be well applicable to investigate further the possible phylogenetic role and the regulation of *POU5F1 *gene.

## Background

The *POU5F1 *gene belongs to the POU (Pit-Oct-Unc) family of transcription factors, that encodes the octamer-binding transcription factor-4 (Oct4) [1]. In mouse, before the zygotic gene activation the active *POU5F1 *mRNA is present in the oocyte. The zygotic expression is activated around the 4-cell stage and later restricted to the pluripotent cells of the inner cell mass (ICM) and to the epiblast. Following implantation, the expression is down-regulated and limited to the primordial germ cells (PGC) but silenced in all somatic cells [2]. *In vitro *embryo studies in bovine, pig, rhesus monkey and in human have shown that the protein is present in the trophoblast cells of blastocyst stage embryos and it is not restricted to the pluripotent ICM cells [3-6]. *In vitro*, *POU5F1 *is highly expressed in human and mouse ESCs. These cells lose their pluripotency during differentiation, therefore the *POU5F1 *expression is diminished [7,8].

The POU5F1 protein is among the core group of transcription factors that induces and controls stemness in ESCs. It sustains pluripotency through feed-forward and feedback transcriptional mechanism and it also plays a crucial role in the early mammalian development [7,9].

The expression of *POU5F1 *is controlled by cis-regulatory elements, located 5' upstream from the initiation site of transcription [2,10]. The regulatory region of *POU5F1 *is highly conserved among species; and usually contains four conserved regions (CR) within the promoter. The TATA-less minimal promoter (MP) region is always located within the first conserved region (CR1) of the upstream sequence of the gene. This minimal promoter contains further primary regulatory elements, such as Sp1/Sp3, and hormone responsive element (HRE) binding sites. Reporter gene expression experiments in mouse with LacZ revealed that two elements, the proximal enhancer (PE) and the distal enhancer (DE) are essential in the cell-specific regulation of *POU5F1*. The proximal enhancer (PE) is located about 1.2 kb upstream, within conserved regions (CR2 and CR3) and is responsible for *POU5F1 *expression in embryonic ectoderm and mouse embryonal carcinoma (EC) cells. Finally, the distal enhancer (DE) located about 2 kb upstream, also within a conserved region (CR4) and drives the *POU5F1 *expression in the morula, ICM, ESC, embryonic germ (EG) and PGC cells of the mouse [2,11,12].

Rabbit (*Oryctolagus cuniculus*) is a classical experimental animal model due to its physiological and immunological properties; it is preferentially used in pulmonary, cardiovascular and metabolic studies, as well as for antibody production and drug screening. In mouse, a wide range of genetic methods have been developed so far, and the successful application of the same technology in rabbits would be very desirable. Recent publications about generating rabbit ESCs [13,14] and successful somatic cell nuclear transfer (SCNT) experiments [15,16] raise the possibility for increasing the use of rabbit model systems for wider applications.

Although the rabbit *POU5F1 *cDNA and the genomic DNA sequences were recently published [17], the isolation and the detailed characterisation of its regulatory region is still missing. Moreover, its expression during embryo preimplantation has not yet been well characterized [18]. This study for the first time describes isolation and sequencing of the rabbit orthologue of *POU5F1 *5' regulatory region and the identification of its phylogenetically conserved regions. We also examined the function of the individual truncated promoter regions using GFP reporter gene system. In addition we also examined the expression pattern of rabbit *POU5F1 *in individual preimplantation stage embryos using quantitative real-time PCR.

## Results

### Isolation of the rabbit POU5F1 upstream region

Recently, the MHC region of rabbit was mapped [19] and partial *POU5F1*-like gene has been sequenced on BAC clones LBAB-841A3 and LBAB-85810. Based on multi-species sequence comparison (human, mouse and bovine *POU5F1 *genes), primers were designed for the conserved regions (oct4-435 and oct4-186, Table 1), and applied for PCR screens and further sequencing.

**Table 1:** Primers used in BAC library screen, promoter analysis and real-time RT-PCRs

**Primer name**	**Primer (5'-3') sequence**	**Position**	**Primer size (bp)**	**Tm (°C)**
**oct4-435-F**	AGCTTAGCTTCAAGAACATG	+3665	20	60.0
**oct4-435-R**	AGGAGTACAGTGCAGTGAAG	+4704	20	60.0
**oct4-186-F**	AGCAGAAACCCTCGTGCAGG	+3759	20	60.0
**oct4-186-R**	TCTGGCGCCGGTTACAGAAC	+4181	20	60.0
*oct4-pseudo-F	GCTAAACAGAAAGAAGTTTGCC	* +420	22	57.0
*oct4-pseudo-R	GAACAGTCACTGCTTGATCGTTT	* +870	23	58.1

**oct4-cDNA-F**	GCTCTACAGAAAGAACTCGAGCAG	+3640	24	57.9
**oct4-cDNA-R**	CGAGTACAGGGTAGCAAAGTGAG	+4869	23	60.0

**CR1-ATGmut**	TCTATGGGGGAAGGAGGGCG	+5	20	60.0
**CR1**	AGGCTGGTGGCATAAAACAC	-558	20	60.0
**CR1+(CR2+CR3)**	GCCAGACTAGAGCCCAACAG	-1585	20	60.0
**CR1+(CR2+CR3)+CR4**	GGGAAATTGTGGAGGAGGAC	-2030	20	60.7
**CR4**	TCTGTCTGCTTGGTGGTGTC	-1670	20	59.9

**Rabbit-oct4-RT-F**	CGAGTGAGAGGCAACTTGG	+4418	19	55.5
**Rabbit-oct4-RT-R**	CGGTTACAGAACCACACACG	+4730	20	56.1
*Rabbit-oct4p-RT-F	CTAAACAGAAAGAAGTTTGCC	* +421	21	53.7
*Rabbit-oct4p-RT-R	CGCAGCTTACACATGTACT	* +594	19	52.3
**Mouse-oct4-RT-F**	ATGCCGTGAAGTTGGAGAAG	+343	20	56.5
**Mouse-oct4-RT-R**	GGTCTGGCTGAACACCTTTC	+3309	20	55.9

The initial ATG is signed as +1, the positions show the genomic locations of the primer. F: forward, R: reverse * pseudogene-specific primers, the positions show the pseudogene location of the primers

The sequenced 7.5 kb *POU5F1*-like region was confirmed as the rabbit's entire *POU5F1 *gene by multiple sequence comparison. Five highly conserved regions have been found, which perfectly matched with the well-characterized five exons of the reference sequences. The lengths of the sequences of these conserved, putative exonic regions were quite similar but not identical, indicating the existence of non-conserved intronic regions. The complete rabbit *POU5F1 *sequence is now referred to as EF194086 and mapped to the MHC region, 12q1.1 [17].

To confirm the coding sequence of *POU5F1*, RACE PCR was applied on blastocyst samples. The gene-specific inner primers were bound to the conserved region of the gene; the outer primers were annealed to the 5'UTR and the polyA tail. The expressed and coding region was found to be 1083 bp, which matched exactly with the previously predicted exons. The rabbit cDNA sequence was identical to human, mouse and bovine in 84%, 83% and 87%, respectively. The cDNA sequence is now referred as EF062856[17].

### Sequence analysis of the rabbit *POU5F1 *promoter region

**Figure 1:**
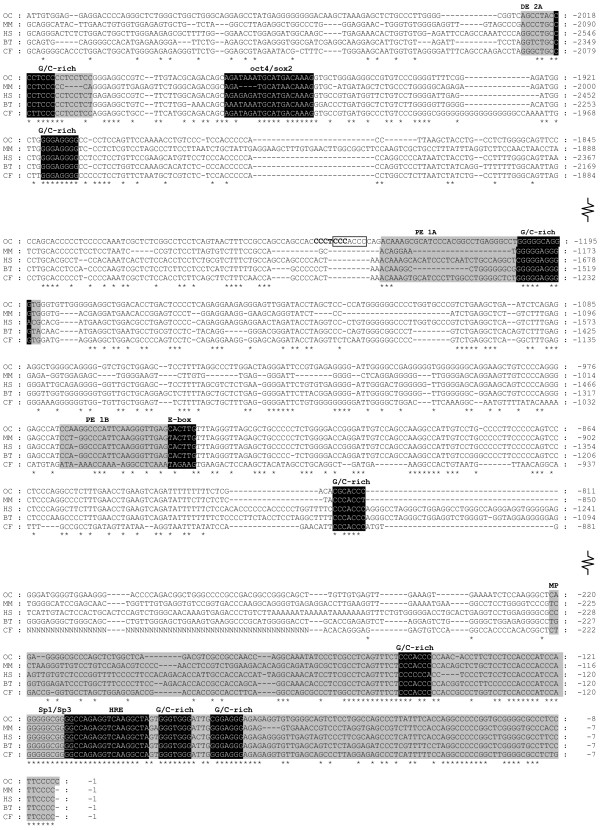
**Alignments of the rabbit (*OC*) *POU5F1 *regulatory regions with its mammalian orthologues**. The region of distal enhancer, site 2A (DE 2A); proximal enhancer, sites 1A and 1B (PE 1A, PE 1B) and the minimal promoter (MP) are shaded in grey colour. The highly conserved GC-rich motifs like GGG(A/T)GGG, CCC(A/T)CCC and the putative transcription factor binding sites are shaded in black colour. A CCCACCC motif near PE 1A present only in rabbit is rimmed; this motif partially overlaps a CCCTCCC motif (bolded). The Sp family binding site is underlined. Nucleotides have been numbered relative to the translation start site of the rabbit *POU5F1 *gene. *Oc, Oryctolagus cuniculus; Mm, Mus musculus; Hs, Homo sapiens; Bt, Bos taurus, and Cf, Canis familiaris*.

**Figure 2:**
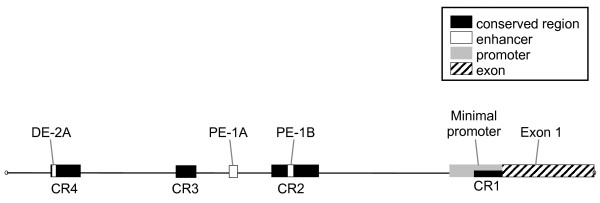
**Organization of the *POU5F1 *promoter region in rabbit (2641 bp)**.

Following confirmation of the *POU5F1*-like sequence as a rabbit coding sequence, the 2.2 kb fragment of the *POU5F1 *upstream region was sequenced and aligned to its orthologues (Figure 1). The alignment proved the presence of four extensively conserved regions (CR1-4) and three enhancer regions in the rabbit *POU5F1 *5' upstream sequence. These CR regions are known to be the functional regions of the *POU5F1 *promoter in several species [11,20]. The different regions in rabbit showed extensive identity compared to the human, bovine, dog and to mouse sequences (Figure 1). In rabbit, the TATA-less minimal promoter (MP) that is located within the CR1 region, extends until -221 bp, and contains both the overlapping binding sites of Sp1/Sp3 (-120/-110), the hormone responsive element (-111/-94) and three G/C rich regions (Figure 1). The E-box sequence in rabbit (CACTTG), that plays a regulatory role in the control of transcription by binding basic helix-loop-helix (bHLH) transcription factors, is located within the proximal enhancer Region 1B (PE-1B;-945/-940). The proximal enhancer Region 1B (-968/-940) is localized within the CR2 region (-1023/-824), while the proximal enhancer Region 1A (PE-1A;-1232/-1192) is located outside of the CR2 region (-1480/-1376). This PE-1A region exceptionally shows less similarity to all reference species. The CR3 region is localized upstream of PE-1A sequence. The distal enhancer region (DE-2A) is located within the CR4 region (-2450/-1901), last from -2025 to -2004 bp. Transcription factor (TF) binding site, Oct4/Sox2 is located (-1977/-1962) in the distal enhancer, within the CR4 region. We have identified several G/C-rich motifs present in all of the five mammalian sequences; however the one near the PE-1A enhancer we found a CCCACCC motif present only in rabbit. This motif partially overlaps the CCCTCCC sequence. The schematic draw of organization of rabbit *POU5F1 *promoter region is shown in Figure 2.

Table 2 shows the pairwise comparison of the rabbit and four other mammalian *POU5F1 *regulatory regions. The analysis unambiguously revealed that the human has the closest potential evolutionary orthologue of the rabbit *POU5F1 *regulatory region. This is supported with the highest homology that could be observed between rabbit and human in all of the four conserved regions and in the PE-1B and DE-2A enhancers. The complete minimal promoter (0/-221 bp) and PE-1A enhancer were the two exceptional regions where the dog sequence showed the highest homology compared to the rabbit sequence (Table 2).

**Table 2:** The homology (%) between the nucleotide sequences of conserved regions and the complete upstream region

**Conserved region of *Oc***	***Mm***	***Hs***	***Bt***	***Cf***
CR1	82.3	**88.4**	86.8	84.8
CR2	92.0	**93.9**	90.7	-
CR3	79.6	**80.9**	73.8	68.2
CR4	67.6	**83.6**	78.0	67.1
PE 1A	76.5	73.2	77.8	**83.3**
PE 1B	96.0	**96.4**	**96.4**	-
DE 2A	87.5	**95.0**	90.0	85.7
MP	74.2	76.8	79.1	**79.3**
5' region	52.5	**57.7**	57.0	51.9

Pairwise comparison method was used to compare the *POU5F1 *regulatory regions of the rabbit and of the four mammalian species. The largest homologies of the regions are bolded; *Oc, Oryctolagus cuniculus; Mm, Mus musculus; Hs, Homo sapiens; Bt, Bos taurus, and Cf, Canis familiaris*; (-) no data.

### Functional analysis of the promoter regions

**Figure 3:**
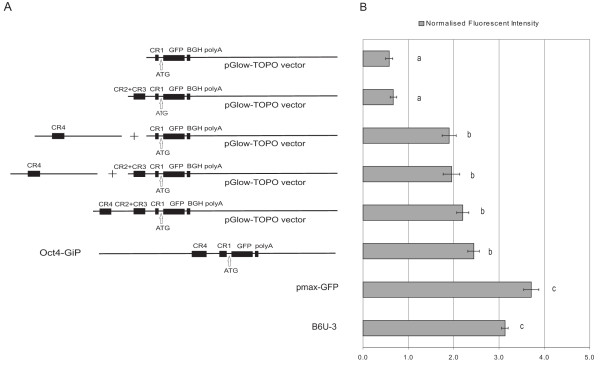
**Expressional analysis of rabbit *POU5F1 *promoter region in mouse ES cells**. A. Schematic view of different constructs used in the assay. B. Fluorescent activity was measured 72 hours post-nucleofection. Data was normalised with the auto-fluorescence of untreated R1 ESCs. CR1, the minimal promoter showed a weak level of basic expression in the mouse ESCs. When the proximal enhancer fragment (CR2 and CR3) was part of the regulator region the expression showed no significant change. However, when the vector containing the distal enhancer region (CR4) was co-transfected either with the minimal promoter (CR1) or with the minimal promoter and proximal enhancers (CR1+(CR2 and CR3), the expression level increased significantly. While the entire regulatory region (CR1+(CR2 and CR3)+CR4) was transfected, the expression peaked to its highest level. If compared to the Oct4-GiP cells containing the mouse CR4+CR1 region, no significant differences were found [20]. S.E. ± values are marked on each column. Different letters mark significant differences between values (P < 0.05).

Four different reporter constructs were electroporated into mouse ESCs to test the functionality of the predicted conserved region in the *POU5F1 *promoter (Figure 3). The minimal (proximal) promoter (CR1), the proximal promoter with proximal enhancers (CR1+(CR2 and CR3), the proximal promoter with distal enhancer (CR1+CR4) and the entire promoter region (CR1+(CR2 and CR3)+CR4) were tested. The fluorescent activity of the minimal promoter (CR1) was very weak compared to the control ubiquitin promoter and to the basic auto-fluorescence of non-transfected control R1 ESCs (Figure 3). Therefore, the rabbit *POU5F1 *regulatory region is capable to express in mouse ESC as a minimal promoter. The addition of CR2+CR3 regions did not increase the level of expression substantially compared to the CR1 fragment. However, when the CR4 region was co-transfected with the minimal promoter (CR1), the level of expression was increased more than two fold. Similarly, the expression was also two fold higher compared to that of the CR1 alone, when the full promoter region [CR1+(CR2 and CR3)+CR4] was transfected or the region of CR1+(CR2 and CR3) was co-transfected with the CR4. However, the intensity of the expression was significantly less compared to the ubiquitin promoter (B6U-3 ESC) or to the control pmaxGFP vector expression (Figure 3). When its expression was compared to the mouse *POU5F1 *minimal promoter (CR1) and distal enhancer-containing promoter region construct (Oct4-GiP) [21], the later exhibited a lower expression intensity in average (Figure 3).

### POU5F1 expression in preimplantation stage embryos

**Figure 4:**
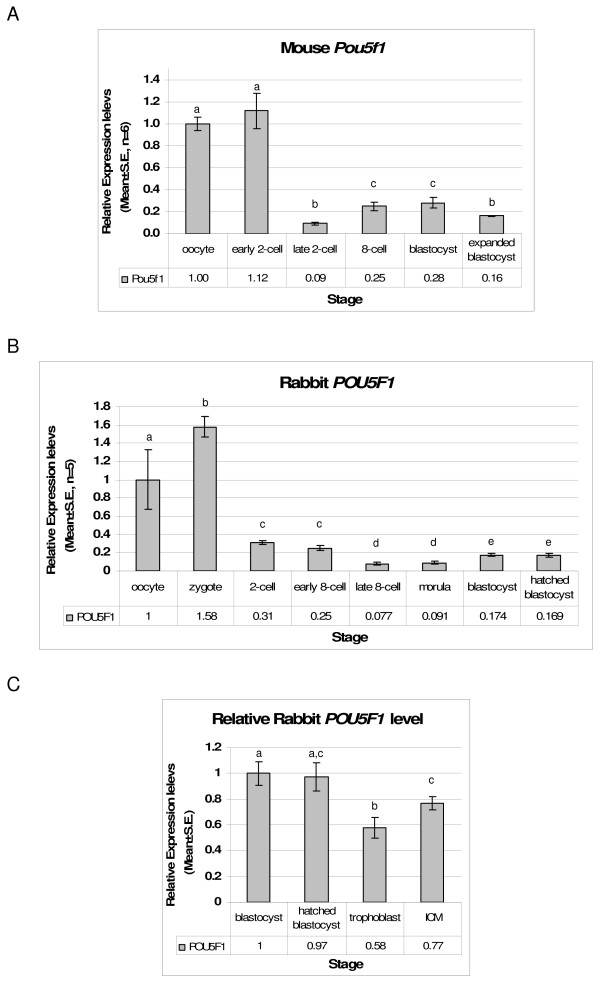
**Oct4 expression in preimplantation stage rabbit embryos**. A. Quantitative real time RT-PCR analysis of *POU5F1 *expression in mouse and B. rabbit preimplantation stage embryos. C. Relative expression level of *POU5F1 *in ICM and trophoblast portion of rabbit blastocyst. The mean values of gene expression of six individual samples and ± S.E. are shown on the diagrams. For comparison of expression through developmental stages (B), the oocyte level was taken as calibrator, while for comparison of TE and ICM samples (C), the blastocyst level was taken as calibrator. Note the scale of the y-axis differs in the case of the two species. Different letters mark significant differences between values (P < 0.05).

The expression of rabbit *POU5F1 *gene was analyzed by quantitative real-time RT-PCR in preimplantation stage rabbit embryos, and compared with the mouse POU5F1 expression. In all examined stages the mRNA of *POU5F1 *was detectable in the embryos with various intensities. The mRNA was present in oocytes and zygotes at a higher level (Figure 4a and 4b), but the levels continuously decreased until the embryonic genome activation, which occurs between late 8- to 16-cell stages during rabbit preimplantation development [18,22,23]). Following the embryonic genome activation, the expression levels increased continuously until the blastocyst stage (Figure 4b). The POU5F1 transcript was also quantified in the isolated ICM and trophoblast portions of the rabbit blastocysts and the relative levels were compared taking the blastocyst transcript as calibrator. The comparison revealed that the *POU5F1 *gene was clearly expressed in both cell types but at a significantly higher (p < 0.05) level in the ICM compared to the trophoblast cells (Figure 4c).

### Identification of pseudogenes

BAC library screen was performed with primers oct4-435 and oct4-186 (Table 1) and BAC clones LBAB-304A07 and LBAB-779H10 were identified. Both clones contain a *POU5F1*-like sequence, and although they lack introns, they contain remnants of polyA tail flanked by direct repeats, all of the characteristics of retrotransposed pseudogenes [24,25]. Based on sequencing the flanking region, the two BAC clones were found to be identical, containing the same genomic region. Yet, the chromosomal localization of these BAC clones is unknown.

The cDNA of *POU5F1 *gene and its pseudogene share a 99% overall sequence identity [see Additional file 1]. Sequence analysis revealed that there is an eight nucleotide deletion in the pseudogene in position 796 bp from the initial ATG that causes a frameshift in the sequence. This results the encoding of a 162amino acid-long truncated protein instead of the 360 amino acid-long functional protein. This truncated form lacks the functional POU domain, but still has a 92% similarity on amino acid level until the frameshift caused STOP codon. The sequence of the pseudogene is referred as EU191070.

Furthermore, to test whether the pseudogene was retrotransposed back near to a functioning promoter, we examined its expression using pseudogene-specific primers in RT-qPCR experiment. The analysis showed that the pseudogene is not expressing at any preimplantation developmental-stages in rabbit [see Additional file 1].

## Discussion

This is the first study describing the cloning and the analysis of the complete regulatory region of the rabbit *POU5F1 *gene. We have also cloned the rabbit Pou5f1 cDNA and analyzed the expression pattern in preimplantation-stage embryos. In addition, we identified a processed pseudogene, having a high degree of similarity.

Our results demonstrate that both the gene structure and the sequence of *POU5F1 *are extremely conserved among various species (rabbit and its murine, human, bovine and dog counterparts). As in other species, *POU5F1 *was mapped to the MHC region, and contained 5 exons with a longer first intron and 3' UTR. The putative protein sequence is almost identical with that of other species. This indicates the highly conserved role of *POU5F1 *in early vertebrate development.

In our study, the 2.2 kb fragment of the rabbit *POU5F1 *upstream region was isolated and aligned to its orthologues (Figure 1; GenBank: EU189135). Comparing the 5' region of human, bovine, mouse and dog *POU5F*1 sequences to the rabbit, all the functionally important regions could be unquestionably defined within 3 kb upstream of its transcription start site. In comparison to the mouse, the rabbit sequence lacks large intersections between CR1 and CR2 regions, but the functional regions in all the four species were almost identical. Several conserved repetitive sequences, E-boxes, the binding site of Pem, Pax-3 and Sp1 transcription factors [26] were identified. Furthermore, the HRE binding site was also identified which was shown previously to be important in RA-induced *POU5F1 *down regulation. The newly identified CCC(A/T)CCC motifs that we have identified only in rabbit might gain importance when studying the differences of *POU5F1 *gene regulation in mammalian species.

The CR4 region is of particular interest for pluripotent stem cell research [21], because this region is responsible for the *POU5F1 *expression in ESCs [2]. Previously, the extended comparative sequence analysis of other mammals (pig, dog, rat) clearly showed three highly consensus sites inside the CR4 region [27]. In rabbit, all three sequences are identical; this suggests the functional significance and a highly conserved regulatory role in mammals. Using comparative sequence analysis we observed that the rabbit and the human *POU5F1 *regulatory regions share the greatest homology among the investigated four species.

Our functional assessment studies on mouse ESCs verified the sequence analysis results. In our experiments the rabbit CR4 region containing the promoter fragments showed the highest expression level in mouse ESCs. This suggests that the rabbit CR4 promoter region confer the same transcriptional regulation activity as the mouse CR4 sequence in mouse ESCs. Also, the rabbit construct showed no significant difference in expression intensity compared to the mouse *POU5F1 *promoter (Oct4-GiP).

The quantitative real-time RT-PCR showed that there is a high level of maternal origin *POU5F1 *mRNA in oocytes and zygotes, which decreased gradually until 8 to 16-cell stage. The transiently reduced transcript levels increased continuously after the embryonic genome activation (Figure 4b). These data are in agreement with the measured (Figure 4a) and published gene expression patterns of mouse [28]. However, since the embryo genome activation in rabbit starts later than in mouse, a difference in the timing of endogenous *POU5F1 *expressions was detected between mouse and rabbit.

In mouse, the expression becomes confined to the ICM following the morula stage, up-regulated in the primitive ectoderm, and eventually becomes confined to primordial germ cells [2]. In rabbit, we found that the *POU5F1 *was expressed in both the trophoblast and the ICM at the mRNA level. In bovine, porcine, rhesus monkey and human preimplantation stage embryos the *POU5F1 *protein was also localized in both cell types [3-6]. In bovine the presence of *POU5F1 *protein in trophoblast cells might act as a suppressor of extraembryonic lineage-specific gene expression during the preimplantation development which allows an extensive proliferation of the trophoblast before the implantation [4,29]. These results indicate that the restriction of expression in blastocyst stage is species-specific [3].

Finally, we have identified a processed, retrotransposed pseudogene of the rabbit *POU5F1*. Retrotransposed genes are generated from mRNA and they integrate back to the genome after reverse transcription. Due to this process, these kinds of pseudogenes usually lack a functioning promoter, or endure some modification, which make them unable to code the functional or full-length proteins. Here we confirmed that the newly identified rabbit *POU5F1 *pseudogene was not expressed during rabbit preimplantation development. Further studies will be needed to clarify its expression in further developmental stages and in differentiated tissues as some pseudogenes are known to possess certain regulatory roles [30-32]. Bioinformatics analysis revealed that retrotransposition frequency of ESC-specific genes appears far to exceed that of non-ESC specific genes, and multiple highly homologous pseudogenes likely exist for all of the ESC-specific genes [33,34]. This is a characteristic of human [35] and also of mouse [1,36,37]. It is not clear yet whether this abundant presence of pluripotency pseudogenes in mouse and human would be also an indication of unknown functions. In human the *POU5F1P1 *pseudogene encodes a putative protein similar to *POU5F1 *isoform 1, which is localized close to the amplified region of a variety of human malignancies [38]. Recent reports on human *POU5F1 *pseudogenes suggested - especially in somatic cells, tissue-specific stem cells, and non-germ cell tumours - that some re-evaluation of expression data on *POU5F1 *might be needed due to the use of primers which cannot distinguish pseudogenes from the coding one [39-41]. In other species - including the mouse and rabbit - the functional relevance of pseudogenes of *POU5F1 *is still uncovered.

## Conclusion

This study provides the first comprehensive analysis of rabbit *POU5F1 *regulatory domains. Detailed sequence analysis of the isolated rabbit *POU5F1 *promoter revealed high sequence conservation in the identified enhancer domains. The strong conservation of regulatory domain sequences shows the importance of the control of *POU5F1 *expression in early mammalian development. We have shown that the highest homology of enhancers can be identified between human and rabbit. This may increase the importance to use the rabbit as a comparative functional genomics tool for human medical research.

## Methods

All chemicals, unless otherwise stated, were purchased from Invitrogen Inc. (Carlsbad, California, USA).

The animal experiments were executed in full compliance with European and Hungarian laws and regulations, and were approved by the Agricultural Biotechnology Center, Animal Experimentation Committee, Gödöllö, Hungary.

### Rabbit BAC library

From a rabbit genomic BAC library [19] four BAC clones were isolated (LBAB-841A3; LBAB-85810; LBAB-304A07 and LBAB-779H10). The clones are stored at the INRA resource centre [42]. The library screening was performed as reported previously [19].

### Cloning and sequencing

The obtained PCR fragments were cloned into pCR2.1-TOPO vector. BAC clones and plasmid DNAs were purified by QIAGEN Plasmid Maxi Kit and QIAprep Spin Miniprep Kit, respectively (QIAGEN). Sequencing was performed in the ABC Sequencing Laboratory with Applied Biosystems BigDye v3.1 Ready Reaction Terminator Cycle Sequencing Kit (ABI) using the ABI PRISM 3100 Genetic Analyser automated nucleotide sequencer.

The genomic sequence of *Oryctolagus cuniculus *5' regulatory region of POU5F1 gene is deposited at the GenBank under accession number [EU189135].

### Comparative genomics

The *POU5F1 *sequence was submitted and referred as [GenBank: EF062856] for cDNA, and as [GenBank: EF194086] for genomic sequence. For multiple alignments the following genomic, cDNA and protein sequences were used: [GenBank: NT_007592.14], [GenBank: NM_002701.4], [GenBank: NP_002692.2] for human; [GenBank: MGI_101893], [GenBank: NM_013633.2], [GenBank: NP_038661.2] for mouse; and [GenBank: NW_001494164.1], [GenBank: NM_174580.1], [GenBank: NP_777005.1] for bovine, respectively.

For the comparative analysis the *POU5F1 *5' upstream sequences of mouse [GenBank: NT_039649.7], human [GenBank: NT_007592.14g] bovine [GenBankAF022986] and dog [GenBank: NC_006594.2] sequences were aligned by using ClustalW2 program [43]. Pairwise comparison of nucleotide sequences was performed on EMBOSS Pairwise Alignment Algorithms program [44].

### RACE-PCR

Rapid Amplification of cDNA Ends (RACE) PCR was performed according to the manufacturer's instruction. As outer primers oct4-5UTR and oligo(dT) primers were used, while oct4-435 and oct4-186 primers were applied as inner, gene-specific primers (Table 1).

### Sample materials

All samples were isolated from Hycole Hybrid rabbits. Oocytes were collected 16 hours post-hCG injection, while one-cell stage embryos were collected 18 hours post-hCG injection from adult female rabbits. Zygotes were cultivated in EBSS media [45] at 38.5°C under 5% CO_2 _in air. After removing the *zona pellucida *with Pronase (Sigma), samples were collected in each developmental stage and kept frozen at -86°C until use. ICM and trophoblast samples from blastocyst were separated using the XYClone laser system (Hamilton Thorne Biosciences) [46]. Briefly, a blastocyst held by two large bore capillaries at the trophoblast region and at the ICM region, respectively, was gently pulled apart while exposed to laser pulses at the area of the trophoblast adjacent to the ICM. When the laser pulses cut through the cells, the two separated parts of the blastocyst were stored separately for further analyses. Due to the visually controlled nature of the procedure, the trophoblast sample is guaranteed to be ICM-cell free, however, the ICM sample might contain a small number of trophoblast cells. Mouse oocytes and *in vivo *embryo samples were collected as described earlier [47].

### Functional analysis of the POU5F1 promoter

The conserved regions of the POU5F1 promoter were identified by multiple sequence alignments, amplified with PCR (Table 1) and subcloned into the pGlow TOPO^® ^TA Expression Kit, containing the GFP reporter gene. The start codon of translation was mutated with PCR-based ATG-mutagenesis (Table 1).

The constructs were nucleofected into mouse R1 ESC by using the AMAXA Nucleofector System and Mouse ES cell Nucleofector Kit (Amaxa Biosystems). Electroporation was carried out based on the manufacturer's protocol using program no. A23. Briefly, 5 × 10^6 ^cells were used for the nucleofection and about 5 μg of plasmid DNA were applied, after copy number normalization of the different constructs. Neomycin selection was started 24 hours post-nucleofection by applying 400 μg/ml G418 on the cultures. For transfection control the Amaxa Nucleofector Kit control vector pmaxGFP was used.

The R1 ESC line [48] (provided by Dr. Andras Nagy; Samuel Lunenfeld Research Institute, Toronto, Canada) was cultured using standard protocols [49]. Oct4-GiP transgenic mouse ESC line, expressing the EGFP reporter gene under the control of mouse *POU5F1 *[CR4+CR1] promoter region was kindly provided by Dr. Austin Smith (University of Edinburgh's Centre for Genome Research, Edinburgh, UK) [21]. The transgenic ubiquitin promoter-GFP expressing ESC line (B6U 3) was isolated from C57BL/6-Tg(UBC-GFP)30Scha/J transgenic mice (Stock Number: 004353; The Jackson Laboratory) in our laboratory (unpublished data).

### Fluorimetric assay

For GFP assays, cells were harvested at 72 hours post-transfection and the fluorescent intensity was measured using the Perkin-Elmer LS-50B Luminescence Spectrophotometer (PerkinElmer Life and Analytical Sciences Inc.; excitation: 375 nm, detection: 510 nm). Fluorescence was corrected for background activity shown by cells transfected with the pGlow-TOPO^® ^vector alone. Cell number variation between individual experiments was corrected measuring the cell protein content (Bio-Rad DC Protein Assay system, BioRad Laboratories).

### Primer design and quantitative real-time RT-PCR analysis

To design *POU5F1 *pseudogene-specific primers, we initially performed a BLASTn [50] search to reveal any rabbit *POU5F1 *pseudogene sequence up to date. This led to the only pseudogene sequence referred EU191070. Based on the sequence alignment of the *POU5F1 *mRNA and its pseudogene [see Additional file 1], we designed a primer to the eight nucleotide deletion region, to avoid amplification of the *POU5F1 *mRNA (Table 1).

The procedures of RNA isolation and cDNA synthesis were done as described in our earlier work [51]. Briefly, the mRNA was extracted from individual oocytes and embryos by Dynabeads^® ^mRNA Direct Micro Kit according to the manufacturer's instructions. DNase treatment (Ambion-ABI) was applied on all RNA samples before cDNA synthesis. Genomic DNA was isolated from rabbit skin by phenol-chlorophorm extraction according to the standard methodology. Complementary DNA synthesis was performed with MMLV Reverse Transcriptase and oligodT primers, using the manufacturer's protocol.

Real-time PCR was performed in Rotor-Gene 3000 Real-time PCR Cycler (Corbett Research, Mortlake, Australia). The reaction mixture consisted of SYBR^® ^Green JumpStart™ Taq ReadyMix™ (Sigma), 300 nM of each primer (Table 1), and cDNA of 0.10 embryo equivalent in a final volume of 25 μl.

The reaction conditions were template denaturation and polymerase activation at 95°C for 2 min followed by 45 cycles of 95°C denaturation for 15 sec, 60°C annealing and extension for 45 sec with a single fluorescence measurement at each cycle. When the reaction was completed, melting curves were plotted to confirm the specificity of the product. Data were normalized based on the sets of reference genes (H2afz, Hprt1 and Ywhaz) [18]. Similar normalization procedures during mouse Pou5f1 transcript quantification were carried out using sets of mouse reference genes are published previously [47].

### Statistical analysis

Data were analyzed using the Chi-square test. P-values less than 0.05 were considered statistically significant.

## Authors' contributions

JK jointly conceived the studies and performed the experimental design, carried out subcloning of promoter regions, performed the functional promoter test in mouse ESCs, and wrote the manuscript. KK carried out the molecular genetic studies and participated in the sequence alignment. ZP prepared *in vivo *and *in vitro *rabbit embryos for the experiments. SM carried out the real-time RT-PCRs analyses, and participated in manuscript preparation. CRG carried out the rabbit BAC library screens and provided the clones. ZT participated in cDNA cloning from blastocyst stage rabbit embryos. IB participated in computational analysis of DNA sequences, pseudogene specific primer design, and qPCRs. ABG participated in primer design, data analyses, and performed statistical tests. KT contributed to the experimental manipulations, MKP gave constructive comments on the study design and drafting the manuscript, AD conceived the studies, supervised the study design, execution, analysis, and approved the final version. All authors read and approved the manuscript.

## Supplementary Material

Additional file 1 **Pseudogene of rabbit POU5F1**. The data provided represent the alignment of rabbit cDNA and pseudogene sequence, and the expression of the pseudogene in preimplantation stage rabbit embryos. Click here for file
