# Supplementary material for: Promoter analysis of the rabbit POU5F1 gene and its expression in preimplantation stage embryos
Source: BMC Mol Biol. 2009 Sep 4;10:88. doi: 10.1186/1471-2199-10-88 (PMC2751759; doi:10.1186/1471-2199-10-88)
Supplement: Additional file 1 — Pseudogene of rabbit POU5F1. The data provided represent the alignment of rabbit cDNA and pseudogene sequence, and the expression of the pseudogene in preimplantation stage rabbit embryos. [file 1471-2199-10-88-S1.pdf]

A

```
pseudo  cagattttaaaaataactcttctcgattatttcaccaggcccccggtgcggggcaccctccttcccc-atggcgg  74
coding  --------------------------ttatttcaccaggcccccggtgcggggcgccctccttcccccATGGCGG  49

pseudo  gacacctggcctcagacttcaccttctcgcccccgccgggcggtggtggcgatgggcctggagggccggagccag  149
coding  GACACCTGGCCTCAGACTTCGCCTTCTCGCCCCCGCCGGGCGGCGGCGGCGATGGGCCTGGAGGGCCGGAGCCAG  124

pseudo  gctgggtggacccctggacctggctgagcttccaaggccctcccggtgggcccgccatcggcccggggttgcac  224
coding  GCTGGGTGGACCCCCGGACCTGGCTGAGCTTCCAAGGCCCTCCCGGTGGGCCCGCCATCGGCCCGGGGTTGCAC  199

pseudo  cgggcgccgaggtgtgggggattcccccgtgtccgccgccgtatgacttctgtggagcgatggcgcactgcaccc  299
coding  CGGGCGCCGAGGTGTGGGGGATTCCCCCGTGCCCGCCGCCGTATGACTTCTGCGGAGCGATGGCGCACTGCGCCC  274

pseudo  cacagcttgccgtgggcctcgtgcctcagggcggcctggagacctctcagccggagggcaaggtaggggccggcg  374
coding  CGCAGCTCGCCGTGGGCCTCGTGCCTCAGGGCGGCCTGGAGACCTCTCAGCCGGAGGGCGAGGCAGGGGCCGGCG  349

pseudo  cggggggcctgtcggaggggccctcccctgagccctgcgctgcgcctcttggtgccgtgaagctggagaaggaga  449
coding  CGGGGAGCCTGTCGGAGGGGCCCTCCCCTGAGCCCTGCGCTGCGCCTCTTGGTGCCGTGAAGCTGGAGAAGGAGA  424

pseudo  agctggagcaaaacccccaaggagtcccaggacatgaaagctaaacagaaagaa--------gtttgccaagctcc  516
coding  AGCTGGAGCAAACCCCCGAGGAGTCCCAGGACATGAAAGCTCTACAGAAAGAACTCGAGCAGTTTGCCAAGCTCC  499

pseudo  tgaagcagaagaggatcactctgggctacactcaggctgacgtggggctcaccctgggggttctccttggaaagg  591
coding  TGAAGCAGAAGAGGATCACTCTGGGCTACACTCAGGCTGACGTGGGGCTCACCCTGGGGGTTCTCTTTGGAAAGG  574

pseudo  tgttcagccaaaccaccatctgccgattcgaggccctacaactcagtttcaagtacatgtgtaagctgcggcccc  666
coding  TGTTCAGCCAAACCACCATCTGCCGATTCGAGGCCCTACAACTCAGTTTCAAGAACATGTGTAAGCTGCGGCCCC  649

pseudo  tgctgcagaaatgggtggaggaggccgacaacaatgagaaccttcaggagatttgcaaagcggagacccacgtgc  741
coding  TGCTGCAGAAATGGGTGGAGGAGGCCGACAACAATGAGAACCTTCAGGAGATTTGCAAAGCGGAGACCCTCGTGC  724

pseudo  aggcccggaagagaaagcgaacgaatatcgagaactgagtgagaggcaacttggagaacatgttcttgcagtgcc  816
coding  AGGCCCGGAAGAGAAAGCGAACGAGTATTGAGAACCGAGTGAGAGGCAACTTGGAGAACATGTTCCTGCAGTGCC  799

pseudo  cgaaacccacgctgcagcagatcagccacatcgcccagcagctggggctcgagaaagacgtggtccgtgtgtggt  891
coding  CGAAACCCACGCTGCAGCAGATCAGCCACATCGCCCAGCAGCTGGGGCTCGAGAAAGACGTGGTCCGTGTGTGGT  874

pseudo  tctgtaaccggcgccagaagggcaaacgatcaagcagtgactgttcccagtgagaggattttgaggccgccggct  966
coding  TCTGTAACCGGCGCCAGAAGGGCAAACGATCAAGCAGTGACTGTTCCCAACGAGAGGATTTTGAGGCCACCGGCT  949

pseudo  ctcccttcgcagagaggcctatgtcttttcctctggcaccggggtcccatttcagtaccccaggctatggcagcc  1041
coding  CTCCCTTCGCAGGGGGGCCTATGTCTTTTCCTCTGGCACCAGGGCCCCATTTCGGTACCCCAGGCTATGGCAGCC  1024

pseudo  ctcactttcgctaccctgtactctccaatgcccttccctgagggggaagccttcccctctgtgcctgtccctgctc  1116
coding  CTCACTTTTGCTACCCTGTACTCGCCAATGCCCTTCCCTGAGGGGGAAGCCTTCCCCTCTGTGCCTGTCCCTGCTC  1099

pseudo  tgggctcccccatgcattcaaactgagctgcctgtccttcccaggaacccggggtggggacagaggtcgggggta  1191
coding  TGGGCTCCCCCATGCATTCAAACTGAgctgcctgcccttcccaggaacccggggtggaggcagaggtcagggg-a  1173

pseudo  aacgctagggagagagaacctggagtcagggctttggggattcagttcttatttcactatggaaggcattggaaa  1266
coding  gacgctagggagagagaacctggagtcagggctttggggattcag---ttatttcactatggaaggcattggaaa  1245

pseudo  cacaaagggtggggcaaggggttttggaaactggttggcgggaaggtgaagttcaatgatgctcttgattttaat  1341
coding  cacaaagggtggggcaaggagttttggggaactggttggtgggaaggtgaagttcaatgatgctcttgattttaat  1320

pseudo  ccccacatcactttgtttttaaataaagaagcctgagacacaatgaaaaaaaataactctttt  1404
coding  ccccacatcactttgtttttaaataaagaagcctgagacacaacgg-----------------  1366
```

B

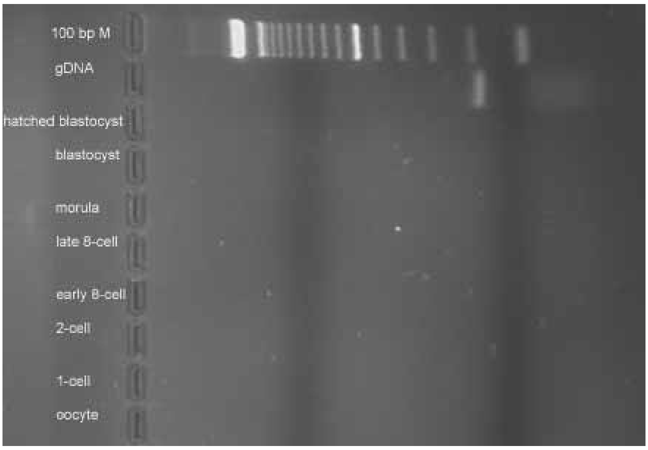

Additional file 1: Pseudogene of rabbit POU5F1.
A) Alignment of the rabbit cDNA and the pseudogene sequence. The coding sequence of the cDNA is upper cased, while the UTR region is lower cased, the initiation site is indicated by an arrow. The pseudogene is flanked by direct repeats in both 5' and 3' direction (underlined upper case). The deletion in the pseudogene sequence is circled. B) The expression of the pseudogene in preimplantation stage rabbit embryos using pseudogene-specific primers in RT-qPCR reactions. The analysis showed that the pseudogene is not expressed in any of the preimplantation -stages of rabbit embryos , although signal is detected with the expected fragment size from gDNA of rabbit, isolated from liver tissue. qPCR products were analysed by running the samples on 2% agarose (1xTAE) gels, containing 0.5 mg/ml ethidium-bromide.
